# Dehydroepiandrosterone-Sulfate, Insulin Resistance and Ovarian Volume Estimation in Patients With Polycystic Ovarian Syndrome 

**Published:** 2017-03

**Authors:** Chrysi Christodoulaki, Eftihios Trakakis, Vasilios Pergialiotis, Periklis Panagopoulos, Charalampos Chrelias, Dimitrios Kassanos, Dimos Sioutis, Nikolaos Papantoniou, Dimitrios Xirofotos

**Affiliations:** The Third Department of Obstetrics and Gynaecology, Attiko Hospital, National and Kapodistrian University of Athens, Athens, Greece

**Keywords:** DHEA-S, PCOS, Ovary, Androgens, Ovarian Volume

## Abstract

**Objective:** To investigate the potential association of DHEA-S with metabolic and hormonal alterations and with disorders of ovarian morphology.

**Materials and methods:** The present study was based on women with PCOS that attended the Gynaecological Endocrinology – Paediatric and Adolescence Endocrinology Department of our clinic. Overall, 321 patients who met the Rotterdam ESHRE/ ASRM – Sponsored criteria for the definition of PCOS were included. Women’s personal medical history was recorded, anthropometric parameters were assessed and blood was drawn for analysis of metabolic and hormonal parameters. A gynaecological ultrasound was also performed to evaluate ovarian morphology.

**Results:** Correlation analysis revealed a significant negative correlation of DHEA-S with the mean volume of the right and left ovary and with the maximum volume of the largest ovary. This finding remained significant after adjusting for age and BMI (β ± SE = -0.39 ± 0.17, p = 0.023 in the case of mean ovarian volume and β ± SE = -0.36 ± 0.17, p = 0.032 in the case of the maximum volume of the maximum ovarian volume).

**Conclusion:** The findings of our study reveal a clear negative association of DHEA-S with ovarian volume. To date, however, current evidence in this field are restricted to experimental animal models. Future clinical studies are needed in this field to corroborate our findings.

## Introduction

Polycystic Ovary Syndrome (PCOS) is the most common endocrine disorder in reproductive age women with an incidence that ranges between 6- 17% (1-3). Its diagnosis is based on the presence of at least two out of the three following features: oligo or anovulation, clinical and/or biochemical hyperandrogenism and polycystic ovarian morphology on ultrasound evaluation (4, 5). PCOS is correlated with a diposity and insulin resistance

(6, 7). Insulin resistance leads to hyperinsulinemia which triggers an overstimulation of ovarian theca cells, that concurrently, leads to increased androgen secretion and decreased production of sex hormone binding globulin (8, 9).

Ovaries and adrenal glands share the same pathway of steroid hormone production. Dehydroepiandrosterone (DHEA) and especially its sulphate ester (DHEA-S) are mainly produced in the adrenal glands (90% of the total circulating amount). Although, approximately 40-70% of women with PCOS have increased levels of Δ4-Androstenedione and DHEA-S, the exact mechanism that triggers the production of androgens from the adrenal gland still remains unclear (10, 11). The correlation between DHEA-S with insulin resistance and glucose metabolism has not been thoroughly examined. Buyalos et al showed that acute induced hyperinsulinemia levels did not affect DHEA-S levels in obese and non-obese women with or without PCOS (12). 

Recent studies have shown that insulin can increase DHEA-S production (13). On the contrary, in other studies DHEA-S is positively correlated to improved insulin resistance in such female population (14). 

Furthermore, DHEA seems to positively influence the antral follicle count and ovarian volume of women undergoing assisted reproduction for primary insufficiency (15). To date however, a clear association of DHEA-S with ovarian morphology in PCOS women has not been proven.

Based on all these data the aim of our study was to investigate the correlation between DHEA-S, insulin resistance, glucose metabolism and ovarian volume in a Greek female population with PCOS.

## Materials and methods

***Patients:*** We designed a prospective study which took place in the Gynaecological Endocrinology – Paediatric and Adolescence Endocrinology Department Outpatient Clinic of the Third Department of Obstetrics and Gynaecology, “ATTIKON” University Hospital between January 2007 and December 2015. All patients with PCOS symptomatology were considered to be eligible for participation in the present study. Initially, 367 patients were screened. The study was approved by the Institution Ethics Committee. After baseline assessment 321 patients who met the Rotterdam ESHRE/ ASRM – Sponsored PCOS criteria were included in the study (4). Patients with a prior history of underlying conditions that could cause hyperandrogenemia or ovarian dysfunction (congenital adrenal hyperplasia, Cushing syndrome, malignancies or medication, thyroid disorders and hyperprolactinaemia) were excluded. Patients who refused to participate in the present study were also excluded. 

A detailed medical history was obtained from all patients. Relevant data regarding age, menstrual cycle, oligo or anovulation or other menstrual disorders, subfertility history, concomitant medical disorders (e.g. thyroid or adrenal diseases, diabetes mellitus etc.), medication (e.g. COCs, hormones etc.), nutrition and lifestyle habits (e.g. smoking, alcohol consumption) and family medical history was recorded.

Subsequently, the patients were clinically examined and their height, weight and body mass index (BMI) was measured. Additionally, clinical signs and symptoms of hyperandrogenemia such as hirsutism, acne, adiposity and androgenic alopecia were assessed. In the case of hirsutism the Ferriman-Gallwey score was used to evaluate its severity (16). According to the classification of the modified method hair growth was rated from 0 to 4 (extensive hair growth) in each of nine locations of the body, resulting in a score that variedfrom a minimum 0 up to a maximum score of 36. For the study population, a score of 6or higher was regarded as indicative of androgen excess. 

***Laboratory Assessment:*** Morning fasting blood samples were drawn during the early follicular phase between day 3 and 6 of the menstrual cycle. After centrifugation Follicular Stimulating Hormone(FSH), Luteinizing Hormone(LH), 17-b estradiol (E2), 17OH-progesterone (17OH-P), prolactin (PRL), total testosterone (T), free testosterone (fT), sex hormone binding globulin (SHBG), dehydroepiandrosterone sulphate (DHEAS), Δ4-Androstenedione, cortisol (E), cholesterol (Chol), HDL, LDL, triglycerides (TG), glucose (Glu) and insulin. All women underwent an Oral Glucose Tolerance Test (OGTT) with 75gr glucose to detect impaired glucose tolerance (IGT) and insulin resistance (IR), after a 8-12 h fasting. Impaired insulin tolerance was diagnosed based on the WHO criteria (17). Insulin and glucose levels were tested at 0’, 60’ and 120’. The diagnosis of insulin resistance (IR) was based on the presence of one of the two following criteria: serum insulin levels at 120’ > 100 μIU/ml or serum insulin levels at 120’ ten folds higher than at 0’. Additional indices for the assessment of insulin resistance were also evaluated and these included the Homeostasis Model Assessment for Insulin Resistance (HOMA-IR), Quantitative Insulin Sensitivity Check Index (QUICKI) and Matsuda Index. The calculation formulas for these were the following:

HOMA-IR: Glucose X Insulin/405

QUICKI: 1 / (log(fasting insulin µU/mL) + log(fasting glucose mg/dL))

Matsuda Index:


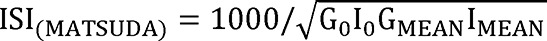


Glucose measurements were performed by the colorimetric method for quantitative measurement of glucose concentration in serum, plasma and urine, (Menarini diagnostics, Firenze, Italy) with coefficients of variance (CV) of 2.33%. Hormone measurements were performed by the ADVIA Centaur Immunoasay System (Siemens, Munich, Germany) for FSH, LH and insulin with CV of 3.9%, 2,7% and 7.5% respectively.

Also measurements of T3, T4, TSH and PRL were performed by the ADVIA Centaur system with CV of 3.44%, 5,55%, 5,87% and 4,8%, respectively. Testosterone, DHEAS and cortisol measurements were performed with the analysis of Elescys 1010/2020 and Modstar analytics E 170 (Roche Diagnostics, Basel, Switzerland) with CV of 5,6%, 6% and 7% respectively. Δ4-Α, 17-ΟHP and free testosterone (FT) measurements were performed with radioimmunoassay kits provided by the Diagnostic Systems Laboratories Inc. (Beckman Coulter, Pasadena, California) with CV of 6,3%, 9,7% and 9,7% respectively. 

***Ultrasound assessment:*** A transvaginal ultrasound examination was performed between day 6 – 8 to assess ovarian morphology, using a General ElectricsVoluson 730 ultrasound machine (General Electrics, Boston, Massachusetts). The scan was performed by a consultant gynaecologist who was specialized in gynecologic ultrasound.

***Statistical analysis:*** Continuous variables are presented with mean and standard deviation (SD) or with median and interquartile range (IQR). Quantitative variables are presented with absolute and relative frequencies. Pearson correlation coefficients were used to explore the association of two continuous variables. The association of DHEA-S with with the mean volume of the right and left ovary and with the maximum volume of the largest ovary was further investigated after adjusting for Body Mass Index (BMI) and age using linear regression analysis. Regression coefficients (β) and standard errors (SE) are presented from the results of regression analysis. DHEA-S, HOMA, QUICKI and MATSUDA were log-transformed due to their skewed distribution and also adjusted for BMI and age. All tests were two-tailed. Statistical significance was set at 0.05 and analyses were conducted using SPSS statistical software (IBM Corp. Released 2010, IBM SPSS Statistics for Windows, Version 19.0. Armonk, NY: IBM Corp).

## Results

Overall 321 PCOS patients were enrolled in the present study. Ten patients requested to be withdrawn from the study in a latter setting. From the remaining, hormonal and biochemical data were available in 303 patients. Τable 1 presents the family history and the anthropometric characteristics of enrolled patients. Descriptive statistics for hormonal measurements are shown in table 2. DHEA-S had a significant and negative correlation with with the mean volume of the right and left ovary and with the maximum volume of the largest ovary (Table 3). The association of DHEA-S with with the mean volume of the right and left ovary remained significant after adjusting for age and BMI (β ± SE = -0.39 ± 0.17, p = 0.023). The same was observed when DHEAS was associated with the maximum volume of the largest ovary (β ± SE= -0.36 ± 0.17, p = 0.032). Correlation of DHEAS with HOMA, QUICKI and MATSUDA was not significant as shown in table 3.

## Discussion

The findings of our study reveal a statistically significant negative correlation between DHEA-S levels mean and maximum ovarian volume (p-values: 0.008, 0.013 respectively). This correlation was further strengthened by them ultivariate analysis when age and BMI were used as independent variables. 

Increased levels of DHEA-S have been previously observed in patients with PCOS and polycystic morphology in ultrasound compared to patients with PCOS without polycystic morphology on ultrasound (18). The negative association of DHEA-S with ovarian volume can be explained by the findings of aprevious experimental study that suggested that PCOS rats treated with DHEA demonstrated ovarian hyperfibrosis which resulted in anovulation (19). According to Miao et al. this effect is triggered through activation of TGF-beta1 and CTGF according to findings of previous studies (20). Rosiglitazone treatment postpones this process. However, it remains unknown if this effect is mediated through DHEA down-regulation.

**Table 1:** Patients’ characteristics

		**n**	**%**
Age, (mean ± SD/median (IQR) (321 patients)		25 ± 5.7	25 (21-29)
Smokers (304 patients)		91	29,9
Family history of menstrual disorders	Free	279	86.9
	Mother	21	6.6
	Sister	2	0.6
	Mother and sister	2	0.6
	Missing data	17	5.3
Family history of hyperandrogenemia	Free	286	89.0
	Mother	14	4.5
	Sister	2	0.6
	Mother and sister	2	0.6
	Missing data	17	5.3
Family history of diabetes mellitus	Free	224	69.8
	Father	43	13.4
	Mother	29	9.0
	Both parents	9	2.8
	Missing data	16	5.0
Family history CAD	Free	231	72.0
	Mother	48	14.9
	Father	19	5.9
	Both parents	8	2.5
	Missing data	15	4.7
Personal history	Free	278	86.7
	Hypothyroidism	21	6.5
	Diabetes mellitus	1	0.3
	Missing data	21	6.5
ΒΜΙ, mean ± SD median (range) (313 patients)		25.7 ± 6.8	23.4 (20.6-30)
ΒΜΙ	Normal	186	57.9
	Overweight	49	15.3
	Obese	78	24.3
	Missing	8	2.5
Menstrual disorders	26-35 days	86	26.8
	> 35 days	180	56.0
	< 26 days	16	5.0
	Amenorrhea	27	8.4
	Missing data	12	3.8
Lorenzo score, mean ± SD/median (IQR)		9.7 ± 4.0	10 (6-12)
Acne	No	133	41.5
	Mild	159	49.5
	Severe	13	4.0
	Missing data	16	5.0
Androgenic alopecia	Yes	187	64.0
Acanthosis nigricans	Yes	292	98.6
Phenotype	Normal	272	100.0
Waist circumference, mean ± SD/median (IQR) (303 patients)		90.4 ± 15.9	88 (78-100)
Hip circumference, mean ± SD/median (IQR) (303 patients)		79.4 ± 28.8	67 (58-106)
Waist to hip circumference, mean±SD/median (IQR) (303 patients)		1 ± 0.4	0.8 (0.7-1.4)
Mean ovarian volume (303 patients)		10.7 ± 4.4	10.25 (7.7-12.8)
Maximum ovarian volume (303 patients)		12.1 ± 5.1	11.4 (8.3-14.4)

CAD: congenital adrenal hyperplasia, SD: standard deviation, IQR: interquartile range

**Table 2:** Hormonal assessment of patients (Outcomes from 303 patients)

		**Mean** ** ± SD**		**Median (IQR)**
FSH (mU/ml)		5.9 ± 2.2		5.6 (4.4-6.8)
LH (mU/ml)		6.4 ± 3.9		5.5 (3.8-7.8)
PRL		18.3 ± 16.9		15 (10.5-21.3)
E2 (pg/ml)		52.2 ± 60.8		39.2 (31-53)
Testosterone (ng/dl)		57.5 ± 25.5		59.6 (39-72)
Free testosterone (pg/ml)		2.2 ± 1.4		2 (1.3-2.7)
OHP17 (ng/ml)		1.3 ± 0.8		1.1 (0.8-1.6)
DHEAS (μg/dl)	238.4 ± 203.4			210 (139-313)
Δ4 Androstendione (nmol/L)			2.6 ± 1.3	2.4 (1.8-3.2)
SHBG (nmol/L)		49.2 ± 34.5		41.8 (28.8-61)
Cortisol (mg/dl)		24.7 ± 35.1		18.3 (12.6-23)
T3 (nmol/L)		3.1 ± 12.9		1.4 (1.1-2)
T4 (μg/dl)		8.6 ± 9.3		7.7 (6.8-8.8)
HOMA		2.8 ± 3		2 (1.2-3)
QUICKI		0.3 ± 0		0.3 (0.3-0.4)
MATSUDA		9.9 ± 6.9		9 (4.8-13)
TSH (mU/L)		2.3 ± 1.6		2 (1.3-2.7)
Negative antithyroid antibodies (%)		50 (86.2)		

IQR: interquartile range

We also investigated the correlation between DHEA-S and HOMA, QUICKI and Matsuda indices, but failed to observe any type of association. These findings could underline the difficulty to determine the role of hyperinsulinemia and insulin resistance in the adrenal and ovarian steroidogenesis and especially DHEA-S secretion. Previous studies in the field suggest that DHEA-S is negatively associated with fasting insulin in PCOS patients (14, 21). Goodarzi et al, however, showed that both insulin and glucose do not influence the adrenal precursor androgen excess which can be found in 20-30% of PCOS patients (22).

**Table 3:** Correlation coefficients of ovarian morphology and indices of insulin resistance

**DHEAS (μg/dl)**		
**Ovarian morphology**		
Mean volume of right and left ovary	r	-0.12
	p	0.023
Maximum volume of right and left ovary	r	-0.15
	p	0.032
Insulin resistance		
HOMA	r	0.006
	p	0.724
QUICKI	r	-0.01
	p	0.651
MATSUDA	r	-0.06
	p	0.392

r = Pearson correlation coefficient

***Strengths of our study:*** The findings of our study are based on a significant number of women that were consecutively recruited on a prospective basis with specific exclusion criteria which limited other potential reasons of DHEA-S excess. For this reason, the possibility of selection bias is low. 

***Clinical implications and future research:*** In our study DHEA-S levels were associated with reduced mean ovarian volume. The exact reason that influences this observation remains unknown; however, previous studies suggest that DHEA administration in experimental models might lead to ovarian fibrosis. Physicians should take in mind this possible association which might become in the future a useful prognostic tool for patients with PCOS. Future studies are needed, however, in the field to corroborate our findings. These should consistently examine all included parameters. Furthermore, basic research in the field should investigate the underlying pathophysiologic pathways which might interconnect DHEA-S to ovarian morphology.

## Conclusion

Our observational study clearly shows a negative correlation between DHEA-S and ovarian volume. To date, however, the exact pathophysiology behind this association remains unknown. Experimental studies suggest that this effect might be associated by increased fibrosis of ovarian tissue, which ultimately results in menstrual disorders and decreased fertility potential. Given the fact that this field remains unexplored in a clinical setting, further studies are needed to reach firm conclusions and to investigate in depth the underlying mechanisms.
